# Provings and Clinical Observations with High Potencies

**Published:** 1892-02

**Authors:** Malcolm Macfarlan

**Affiliations:** Philadelphia


### PROVINGS AND CLINICAL OBSERVATIONS WITH HIGH POTENCIES.

MALCOLM MACFARLAN, M. D., PHILADELPHIA.

(Continued from December number, page 456.)

ARUM-TRIPH.$^{16M}$.

Burning of pharynx and glottis. *Very nervous*, raw feeling in chest, picking his nose all the time, light-headed, sleepy. Smarting at end of penis.

Feels sick, qualmish, giddy, slept unusually sound, eyes dim. Pain under his left short rib.

Helped a minister's sore throat of years standing—in fact, cured him.

Woman could hardly stand on her feet they hurt her so, even the stockings when drawn on gave her pain. Feet feel so bruised; end of her tongue felt sore, round sore spots, as if scalded on the tongue. Sores on throat and tongue, sensation as of an abscess forming high up inside the nasal bone on the right side, discharge of a large crust. Little round, hard pimples all over the skin of the body, legs, arms, and face, about size of a pin-head, feet hurt her so that it made her sick at stomach to walk. Menses, which were absent two months, return. Soreness and pain, as if bruised in the left mamma. *Nightmare.* Just as she gets to dozing feels as if she would smother, starts up frightened. Nervous at night, could not sleep. Distressing hacking cough; mouth very dry; feels as if mucous surface would crack; wants to wet it, not to drink, has to get up in the night to moisten mouth.

ASCLEPIAS-TUB.$^{45M}$.

Produced free perspirations and urinations, without any other effects.

ASTERIAS-RUB.$^{5C}$.

Pimples on side of nose and chin. Provers were women. Cured many cases where there is a disposition to pimples about the chin, mouth, and face, generally occurring at the menstrual period and with young girls.

Neuralgia opposite left molar teeth of lower jaw, sharp, piercing pain like a needle, lacks confirmation. One prover.

### ATROPINE, 6TH DECIMAL.

Given in water every two hours, cured long-standing photophobia and watering of eyes.

### ATROPINE-SULPHATE$^{30}$.

Wonderful effect in relieving pain attending ulceration of the cornea with violent conjunctivitis, sudden and marked cure of photophobia.

Atropine-sulphate 6th x given in water every hour, produced, on the third day, great dimness of vision. Can't see glassware, such as tumblers and bottles; pupils not dilated, pain in and through the eyeball, cannot see to read well. The accommodation is paralyzed. Cannot see to read even when holding the paper far off. Type looks blurred. Previously had acute normal vision. Never wore glasses. These symptoms lasted over a week. Mouth became so parched she could not speak without first moistening it. Patient took the medicine every hour for two weeks.

Atropine-sulphate$^{6}$ in water every two hours, cured long-standing photophobia and lachrymation.

### AURUM-MUR.$^{5M}$.

Legs appear slightly swollen and very tender along the inner side of the tibia, backache very severe.

### IODIDE OF POTASSIUM$^{CM}$.

After being given three weeks for its curative effects, produced choking sensation in the larynx, slight loss of voice, considerable increase in weight, improved appetite.

### IODINE$^{17M}$.

The long internal administration of Iodine high produced in a girl symptoms of asthenopia; spotted and rough skin; husky voice.

### IPECAC$^{25M}$.

Violent spasmodic cough, aversion to food.

Ipecac, and blanket wrung out of hot water to envelop the child, apparently cured a severe case of membraneous croup. Previously the child appeared hopeless.

JASPER$^{5C}$.

Leucorrhœa, aching in sacrum, no appetite, sensitiveness and sensation of weakness in bowels, dull frontal headache.

KALI-BICHR.$^{CM}$.

Redness in throat, irritation, swollen tonsils. A very reliable remedy in diphtheritic sore throat, loss of appetite, with hoarseness.

KALI-CARB.$^{24M}$.

Stiffness back of neck, shooting pains through his chest; shooting pains in muscles of extremities and chest.

KALI-HYD.$^{CM}$.

Larynx feels sore, gums and mouth sensitive; can hardly eat for soreness. Has cured many cases of rupia and syphiloderms. Fluttering sensation at the heart, giddiness; arose from bed, thinking he would be smothered. After curing skin symptoms produced profuse watery discharges from the nose. Later on, discharge thicker, aching pains through both lungs, feels tired and weak, fluttering at heart and nervous.

The high preparations have frequently cured syphilitic ulcerations, especially on the legs, where the low did nothing.

Sharp pains through the right lung, from the nipple backward, hoarse cough, pains through the breast, sighing respiration.

Iod. Potash, highly potentized and in various potencies, have relieved and cured the most persistent or chronic cases of megrim, sick headache; often the cranial bones were sensitive, after the attacks passed off; more or less nausea and weak vision attending the cases. Follows well after Belladonna.

KAOLIN$^{45M}$.

Sore throat, soreness of both lungs, constipation. Has frequently cured cases of constipation, with large, hard, dry, lightcolored stools; great internal soreness of the chest often relieved; painful respiration—not walls of chest, but lungs, apparently.

### KREOSOTE$^{CM}$.

I know of no remedy which can compare with this in cases of cholera infantum, green stools, nausea, exhaustion, complete loss of appetite, dry skin, more or less fever, types one often sees in midsummer in the large cities.

Indigestion, loss of appetite. Caused sick stomach, disposition to vomit.

### LITHIA-CARB.$^{5C}$.

Cured a chronic syphilitic bluish deep old ulcer, larger than a silver dollar, on the calf of the leg in a young man. Had to get up at night to pass water. Frequent urination, with slight pain or straining; pain over the bladder; appeared to dilate pupils. The latter lacks confirmation.

### LACHESIS$^{16M}$.

Has cured for me a great number of cases of chronic sore throat, dryness in throat, often raw or sore, with much swelling. After producing throat symptoms in a young man, it caused severe pains all over his head, back and front. Giddy, couldn't stand, had to be carried from school, couldn't see the letters in his book, fell against the wall, etc. These occurred on the fourth day; never been so affected before.

CM quickly curative in cases of delirium tremens, where there is much trembling and confusion of ideas. Given every one-quarter hour for three days produced frightful constrictive sensation at the larynx, almost suffocated at night. In an attack woke the whole family; they poured a quantity of brandy down her throat with but little effect. All thought she was dying from inability to breathe or expand the lungs. Sat up in bed struggling for breath like an asthmatic.

CM, in a woman at forty-five, produced rolling from side to side in the bed constantly, from hour to hour. Could not stop her; extreme restlessness and nervousness.

Lachesis every quarter-hour apparently cured a case of membraneous croup in last stages. Coughed up pieces like a cast of the bronchus and was relieved in two hours.

6th produced in several young persons very sore throat.

CM has cured repeatedly diphtheritic sore throat and has been of great service in malignant scarlet fever with very offensive breath ; glands of neck swollen ; soreness of neck to touch ; suffocative attacks as soon as anything touches the larynx. Even the front part of the throat is sensitive, has to loosen everything around the neck to breathe better.

CM caused suffocative sensation ; distress on either side of sternum at its middle.

CM cured hoarseness with complete loss of voice very many times.

### LACTUCA-VIROS$^{5C}$.

Right eye smarted a good deal ; pains catch him in his lungs, back and front ; pains across the top of his sacrum.

### LAMIUM$^{1M}$.

Severe sick headaches in the mornings, giddy, worse on left side ; muscles generally sore, as if the prover had taken cold ; muscular and mental exhaustion with no inclination for food.

### LAUROCERASUS$^{CM}$.

CM helped cough and greatly lessened the chronic expectorations in persons of consumptive tendency ; produces a short, dry hack or clearing of the throat in others not previously so affected.

### LITHIA-CARB.$^{5C}$.

Have found it highly curative in barber's itch ; circular moist furfuraceous patches on the skin ; porrigo.

5C causing a rough rash all over the body ; much loose epithelium ; tough, dry, itchy skin, turbid urine.

5C. Skin of the whole body is rough and dry. The face, or rather both cheeks were covered with dry, bran-like scales. This was produced on several infants to whom I had given the medicine for some time.

### LEDUM$^{45M}$.

Gnawing headache in temples ; headache of back of the head and ears ; talking in sleep ; nausea ; confused vivid dreams.

### Lilium-tigrinum$^{45M}$.

Compelled to pass water frequently; with burning sensation; feels bruised about genitals; bowels that were costive move more regularly; smothering sensation in the chest, feels like vomiting when she touches her epigastrium. Feels as if she must cross her legs for fear everything in pelvis would be pushed out. Her head feels confused and heavy, glands or left side of neck slightly swollen and painful.

### Antirrhinum Linaria 30th decimal and lower.

*Fainting* spells occurring three or four times a day; fainted dead away; never occurred before. The prover was rather a weak individual, disposed to phthisis, fainting without any other symptom, attacks of weakness and fainty feeling from probable disturbed heart action, due to the medicine. Frequently verified this. Sensation of fainting.

### Linaria.

Antirrhinum Linarium—Gathered the flowers and made the potencies, 3d to 30th, myself, taken in water, given every hour or two. Giddy, sick stomach, perspiration, rumbling of gas, bowels somewhat loose, vomited several times on fourth day. These symptoms were developed in several women. No urinary symptoms produced as I had expected.

### Lobelia$^{45M}$.

The oppression is felt in the throat. Sick stomach with ptyalism.

### Lycopodium$^{45M}$.

Highly curative in violent gall-stone colic. The attacks were painful while they lasted; medicine generally relieves quickly. Verified this repeatedly.

45M. I believe I am indebted to this medicine (as a means) for the cure of pneumonia in my child when two years old. It has often acted like magic in the relief of bronchitis of young children especially.

45M acts in a wonderful way in curing acute bronchitis of children. Great *rattling of mucus in the chest.* Chest oppressed, breathing rapid, cough frequent and loose.

45M. Child frequently wakes at night and rubs her nose so much and long that the parents are astonished. Have frequently verified this symptom.

45M caused symptoms of *cold in the head*, verified in very many cases.

45M caused sniffles in children. Symptoms of influenza.

45M. Remarkable in relieving the short and painful breathing of pneumonia.

45M given to boy, aged eight years, every two hours for a week, who had scrofulous ophthalmia, caused severe pain in the left side of the chest, breathing oppressed and painful like that of pleurisy.

### MENTHA-PIP.$^{5C}$.

Pharynx inflamed. Produced very sore throat, not swollen, simply dryness and redness; griping pain in median line below the umbilicus (4M); bowels a little loose, simply griping.

### MEPHITIS$^{1M}$.

Cured frequently very severe, hoarse, hollow, deep cough, with soreness in the chest (often verified); convulsive, teasing, tickling cough without expectoration. The medicine causes this cough in those not afflicted with it.

### MERCURIUS-CORROSIVUS$^{5C}$.

Curative in constipation, with so-called bilious condition. Sensation as if the mouth was scalded. Soreness of mucus membrane; frequently curative in bloody, painful diarrhœa of a chronic kind, dysentery, colic, rectal pains.

5C. After trying many remedies, this was the only one which cured bloody, frequent, offensive stools, mixed with mucus, with great pain in the abdomen. This from my army experience and subsequently.

Left testicle swollen, or sensitive in some cases, soreness at sides of the waist, general weakness, no strength to work properly, left hypochondrium worse than the right.

### MERC-PROD-IOD.$^{2C}$

Given to cases of hard chancre caused a number of pimples to appear, changing to pustules resembling small-pox. Verified this in a great number of cases. In children produced frequent green stools. 2C, constant tendency to hawk and spit, frequently cured this symptom with it. 2C, pressive pain in forehead, worse at night, feels as if bruised all over, especially thighs; passes water seldom, but in very large quantities; it soon becomes turbid on standing. Feeling as of a great ball in the epigastrium. Frequently cured the symptom of urine turbid from excess of urates, and being alkaline.

### MERC-VIV.$^{10M}$.

Chilly sensation, with general muscular soreness, mostly in his back; soreness in muscles laid him up for three days.

10M given to a girl six times a day for a week. Great soreness of the thighs to touch; same with the arms, can hardly raise them. Complete loss of appetite. Constipation at first.

Free perspiration in some provers, which does not relieve the soreness.

Sore all over, general shooting pains, can't bear the least noise, back pains him, aching from head to foot, left side of throat more sore than right. Highly curative in several cases of chronic ophthalmia. Great pain in *forehead*, and in *top* and *back* of his head.

Excellent remedy where pus is in the anterior chamber of the eye, judging from my limited experience with it in this trouble. *Merc-corros.* has cured repeatedly scrofulous ophthalmia, disposition to pimples about the face and body like small boils.

10M. Limbs ached all over, mostly on the right side, *espe-*

*cially in the small of the back and at the sides;* right shoulder joint pained him much, limbs sore to the touch, feverish. He is pained over the eyes when they are moved suddenly. Soreness within, from the larynx to ensiform cartilage; does not hurt him to breathe, but swallowing is painful because of swollen tonsils.

### MEZEREUM$^{103M}$.

Highly curative in severe nervous headaches through the temples. Pains shooting through the eyeball to the back of the head. One of the best remedies in violent ciliary neuralgia, either from disease or after operation; relieves the pain in glaucoma, has been given it often with wonderfully good effect.

### MURIATIC-ACID$^{5C}$.

Heat on top of the head, piles, wants to lie abed, so tired; slight neuralgia on left side of head, slightly sick at stomach, some diarrhœa and pain in the bowels.

### MYRICA-CERIFERA$^{45M}$.

Mist before the eyes, appearance as if of a flame before them. Can't see well. This remedy has not been proven enough to place much dependence on the symptoms.

### MYRTUS-COM.$^{5C}$.

Rheumatic-like pain in the armpits and shoulders; severe joint pains. Promptly relieved and cured distress and bearing-down sensation in uterus, with ovarian soreness.

### NATRUM-MUR.$^{CM}$.

Caused buzzing in ears in a number of cases.

CM (Fincke) has cured a great number of cases of fever and ague given in water every two hours or eight times daily.

CM had a *wonderful effect* in restoring vision in chronic asthenopia. Cured symptom of spots in field of vision.

CM caused sores in mouth resembling aphthæ, often verified.

### NITRIC-ACID$^{5C}$.

*Eyes weak*, great soreness along the tibia, periosteum sensitive, had to wrap flannels on the legs to relieve the pain; piercing pain in the temples, sores (a crop of them) all around the mouth like small fever blisters.

Oppressed chest, *pain across both buttocks*, throat slightly sore, gums sensitive, *margin of the mouth covered with sores*, rash over her face and forehead, small pimples, no appetite, vomits occasionally, bowels move twice daily, eyes excessively painful and felt too sore to use much, head feels hot; legs (left more than right) very sore in front from ankle to knee; within the nostrils sore.

Rapidly cured cases of syphilitic ulcerated sore throat, syphilitic white patches in the mouth, and many cases of chronic syphilitic ulcerations in various parts of the body, where Iodide of Potassium had been given without effect.

### NITRUM$^{5C}$.

Disposition to bite the lips; a nervous affection, crackling or clicking in ears.

5C arrested most violent attacks of asthma; relieved spasmodic breathing in heart affections.

5C cured a number of cases of painful menstruation, chronic cases, when the flow is delayed and scanty; uterine colic.

### NUX-MOSCHATA.

It seemed as if the lower limbs were light as a feather; head felt light as if he had taken ether; numbness in the seat and down the thighs; flighty at night; chilly and feverish; diarrhœa; small stools; straining.

### NUX-VOMICA$^{94M}$.

Passes water more freely; cured the burning on voiding it; relieved a dull, severe pain in epigastrium; felt as if the stomach was sore.

Head became clearer, cured a nervous state and sensation of falling when asleep; nervousness; disposition to nightmare; produced great sneezing; slight soreness in the throat and watery discharge from the nose; burning, scalding on passing urine; sore patches on mouth and tongue; *coated tongue;* can't use tobacco in any way; bowels costive (previously regular); gaping frequently, just as if a person were sleepy; confined him to bed; made him very sick; aches from the top to back of head and neck, which feels stiff; vomits his breakfast; has to force himself to eat.

Frequently relieved the ill-effects of eye strain, asthenopia, and slight intolerance of light.

### OLEANDER$^{5C}$.

Sensation of numbness in upper and lower extremities. Have frequently verified this symptom.

### OXALIS$^{1M}$.

Frequent nose-bleed, not accustomed to this; reading affects her head and type becomes blurred; warm flushes; chilly and nervous; *dragging sensations from hip to groin;* feels as if everything in pelvis would be pressed out; urinates freely; pain in kidney so severe as to cause the prover to be bent to find some relief.

### PALLADIUM$^{20M}$.

Wakeful until two o'clock in the morning; headache across the top of his head from one ear to the other.

Pain in head, just above and behind the tip of the right ear.

### PETROLEUM$^{CM}$.

Has cured very many cases of chronic eczema; parts seem excoriated; the knees feel tired; fatigued easily; thirsty; urinates frequently, and bowels disposed to be loose; slight, dry, hacking cough.

### PHELLANDRIUM-AQUAT.$^{5C}$.

Giddy when lying down; nauseated; slight sore throat.

### SOME REMARKS ON HAMAMELIS VIRGINIANA.

Dr. Bree.

All provings of this drug show its remarkable influence on the vascular system, especially in hemorrhages of any organ, as mostly the first or second decimal dilution suffices, without the necessity of descending to the mother tincture. It is often indicated in metrorrhagia after abortus, post-partum or from tumors; but in acute arterial flooding needs other remedies. It acts equally well in dysmenorrhœa, amenorrhœa. In too copious and too long menstruation, the flow and the pains diminish, the fullness of the abdomen disappears, the general malaise gives way to returning health. It is nearly indispensable during climaxis. Not only hemorrhages but congestions to any organ are then a good indication.

Hamamelis plays an important part in varices and varicose ulcers, and here it rivals Carduus-mariæ. Varices diminish in size and with it the pains diminish, the margins of the ulcers look better, granulations more healthy, and with cleanliness no other treatment is needed. Its action in blind or bleeding hemorrhoids is, too, well known. Most patients suffer also from hepatic troubles and are dyspeptic. Abdominal plethora disappears and with it the hemorrhoidal troubles, meteorism ceases, appetite improves, the features clear up and work again becomes a pleasure. Cold feet and tearing pains cease, bowels become regular under the action of Hamamelis alone.

In cardiac troubles its action is astonishing, removing congestions and stasis, and chronic catarrhs of the intestinal and respiratory organs are often greatly relieved by it. Most *patients with heart troubles complain of excessive thirst* by day and by night, and it is astonishing what quantities of fluids such patients use up. Hamamelis has the power to moderate or even to stop this thirst, so that sleep becomes more regular and refreshing, and one may well ask whether the cardiac troubles do not originate from an anomaly in the vascular system and abnormal relations of blood pressure. Mostly these abnormal relations are taken as sequelæ of cardiac defects, and if called early enough Hamamelis might prevent it.

Hamamelis will never produce sleep when nervousness is its cause, but it acts well where anomalies in the vascular system produce it, and here a varicose state gives us a beautiful hint for its application, as it also removes the bad dreams and the mental wakefulness which prevents sleep or allows only unrefreshing cat-naps.

Though highly praised in orchitis, it will only benefit such cases which are complicated with varices, hemorrhoids, tortuous temporal arteries, and other tortuous aspects in diverse parts of the body. The great thirst is hardly found in any other remedy, hence it may be considered a great remedy in adiposity, or rather that fullness of the body found in flabbiness and sponginess of the tissues, dependent on relaxation of the capillaries. Blooming red cheeks may simulate good health, but too often such a dilated capillary system is the cause of such a false plethora. Hamamelis constricts the capillaries, the tissues take on their tension, and the patient feels better by it.

A disposition to chillblains can be removed by taking the drug off and on for a whole year. Urinary difficulties of senility often arise from catarrhs which originated in congenital or acquired anomalies of the vascular system. Headaches, fainting spells of blooming persons are benefited by it, as it at the same time removes the superfluous adipose tissue, diminishes thirst, increases the appetite, and allows refreshing sleep.

Hamamelis urges on a sluggish circulation to do better work, tissue change is thus increased, and blood pressure becomes normal. Yet such a state is often found in many pathological states, febrile or afebrile, and taking this in consideration it may be applicable to many cases where so far it was too often neglected.—*Zeitsdv. d. Berliner ber. hom. Aerzte* x, II. S. L.

